# Society Meetings

**Published:** 1906-04

**Authors:** 


### SOCIETY MEETINGS.

The British Medical Association will hold its seventy-fourth annual meeting at Toronto, August 21, 22, 23 and 24, 1906. The section of obstetrics and gynecology is officered as follows: President, A. H. Freeland Barbour, M.D., Edinburgh; Vice-Presidents, Professor James A. Temple, Toronto, Professor Adam H. Wright, Toronto, Professor Wm. Gardner, Montreal, T. Arthur Helme, M.D., Manchester.

The committee responsible for this section would be greatly indebted to members interested in Obstetrics and Gynecology if they would suggest subjects for discussion in this section, such subjects to be of general and practical interest. The committee would also greatly welcome offers of papers and of pathological specimens for the gynecological and obstetrical part of the museum.

Suggestions of subjects for discussion in the section should be forwarded to the Honorary Secretary resident in London by those members resident in Great Britain, and to the Honorary Secretaries resident in Toronto by members resident in the Colonies.—Frederick Fenton, M.D., 75 Bloor Street, Toronto, Kennedy C. McIlwraith, M.D., 54 Avenue Road, Toronto, Cuthbert Lockyer, M.D., F.R.C.S., 117A Harley Street, London, W., Honorary Secretaries.

---

The Buffalo Academy of Medicine held meetings during the month of March as follows:

*Section of Medicine.*—Tuesday, March 6, 1906, at 8:30 P. M. Program: (a) Therapeutic treatment of pneumonia, George H. Westinghouse. (b) Report of 25 cases of lobar pneumonia treated with antipneumonic serum, G. Tartaro.

*Section of Surgery.*—Tuesday, March 13, 1906, at 8:30 P. M. Program: A consideration of acute hematogenous infections of the kidney, George E. Brewer, New York. Complications with fracture of the long bones of the wrist and ankle, Vertner Kenerson.

*Section of Pathology.*—Tuesday, March 20, 1906. The meeting of the pathological section announced in the program for March 20, 1906, was postponed to a later date.

*Section of Obstetrics and Gynecology.*—Tuesday, March 27, 1906, at 8:30 P. M. At this meeting nominations were received for President and Treasurer for the ensuing year, and for a Trustee for three years. Program: The etiology and treatment of cystocele, J. Riddle Goffe, New York. A collation was served at the close of the meeting.

THE ASSOCIATION OF MILITARY SURGEONS OF THE UNITED STATES will hold its fifteenth annual meeting at Buffalo, September 11, 12, 13, 14, 1906, under the following administration:

*President*—Lieut. Col. Albert H. Briggs, N.G.N.Y., Buffalo, N. Y.

*First Vice-President*—Brig. Gen. Robert M. O'Reilly, U.S.A., Washington, D. C.

*Second Vice-President*—Rear Admiral Presley M. Rixey, U.S.N., Washington, D. C.

*Third Vice-President*—Assistant Surg. Gen. George Tully Vaughan, P.H. & M.H.S., Washington, D. C.

*Secretary*—Major James Evelyn Pilcher, U.S.V., Carlisle, Pennsylvania.

*Treasurer*—Major Herbert A. Arnold, N.G. Pa., Ardmore, Pennsylvania.

*Advisory Board*—Hon. L. M. Shaw, Secretary of the Treasury; Hon. C. J. Bonaparte, Secretary of Navy; Rear Admiral P. M. Rixey, U.S.N.; Hon. Wm. H. Taft, Secretary of War; Gen. Robert M. O'Reilly, U.S.A.; Gen. Walter Wyman, P. H. & M. H.S.

*Executive Council*—The officers of the Association, ex-officio, and Major Jefferson R. Kean, U.S.A., Lt. Col. Nathan S. Jarvis, N.G.N.Y.; Med. Insp. William R. Dubose, U.S.N.; Major Thomas C. Clark, Minn. N. G.; Surg. H. W. Austin, P.H. & M. H. S.; Captain Miles Standish, M.V.M.; in conjunction with the Ex-Presidents, ex-officio,—namely, Col. Nicholas Senn, Ill.N.G., Brig. Gen. J. D. Griffith, N.G.Mo.; Brig. Gen. A. J. Stone, Minn. N.G.; Major Gen. Robert Allen Blood, M.V.M; Brig. Gen. Geo. M. Sternberg, U.S.A.; Brig. Gen. Charles H. Alden, U.S.A.; Colonel John Van R. Hoff, U.S.A.; Surgeon Gen. Walter Wyman, P.H. & M.H.S.

*Standing Committees* — LITERARY COMMITTEE — Lieutenant Colonel Eugene A. Smith, N.G.N.Y, Buffalo, N. Y.; Major Charles F. Mason, U.S.A.; Major Edgar Francis Sommer, Ind. N.G.; Surg. William C. Braisted, U.S.N.; Asst. Surg. Wm. C. Rucker, P.H. & M.H.S.; Captain Vertner Kenerson, N.G.N.Y. PUBLICATION COMMITTEE—Major James Evelyn Pilcher, U.S.V.; Carlisle, Pa.; Surg. C. P. Wertenbaker, P.H. & M.H.S.; Lieut. Col. Charles Adams, Illinois N. G.. NECROLOGY COMMITTEE—Capt. Samuel Cecil Stanton, Ill. N.G., 1040 Sheridan Road, Chicago, Ill.; Surg. James Chambers Pryor, U.S.N.; Brig. Gen. J. B. Edwards, Wisconsin N.G. COMMITTEE ON ARRANGEMENTS—Major General Samuel M. Welch,, N.Y.N.G., Buffalo, N. Y.

*Special Committees*—PUBLIC SERVICE MEDICAL SCHOOL COMMITTEE—Colonel John Van Rensselaer Hoff, U.S.A., Washington, D. C. COMMITTEE ON LEGISLATION—Medical Director Robert A. Marmion, U.S.N., Washington, D. C.; Lieut Col. Edmund C. Brush, O.N.G.; Maj. William C. Borden, U.S.A.; Lieut. Col. Julius F. Henkel, Mich. N.G.; Major Louis L. Seaman, U.S.V.E.; Brig. Gen. Marshall O. Terry, N.G.N.Y.; Col. Joseph K. Weaver, N.G.Pa.; Col. Winslow Anderson, N.G.Cal.; Lieut. Col. Wilbur S. Watson, Conn. N.G.; Col. R. Harvey Reed, N.G. Wyo. COMMITTEE ON INSIGNIA—Major General Otis H. Marion, M.V.M., Boston, Mass.; Brig. Gen. James T. Priestley, N.G.Ia.; Lieut. Col. Edmund C. Brush, Ohio N.G.; Col. R. Harvey Reed, N.G. Wyo.; Captain Thomas Page Grant, Ky. S.G.

